# Prevention of caries and obesity in children with immigrant background in Norway- a study protocol for a cluster randomized controlled trial

**DOI:** 10.1186/s12903-023-03329-9

**Published:** 2023-09-01

**Authors:** Mariam Reda, Abhijit Sen, Manal Mustafa

**Affiliations:** 1 Oral Health Centre of Expertise in Western Norway, Bergen, Norway; 2 Center for Oral Health Services and Research (TkMidt), Trondheim, Norway; 3 Department of Public Health and Nursing, Faculty of Medicine, and Health Sciences, Norwegian University of Science and Technology, Trondheim, Norway; 4 Department of Clinical Dentistry, Faculty of Medicine, University of Bergen, Bergen, Norway

**Keywords:** Clinical trial, Dental caries, Pediatric obesity, Parents, Knowledge

## Abstract

**Background:**

Immigrants are known to experience greater socioeconomic stress and poorer well-being and to suffer more from lifestyle- and diet-related disorders than native populations. There is also evidence that children of immigrant parents are at greater risk of diverse health problems than their host country cohorts. The aim of this study is to apply and evaluate the efficacy of an early life intervention program among parents/children with immigrant background to prevent childhood caries and obesity, thereby improving the children’s general health, oral health, and quality of life.

**Methods:**

This is a study protocol for a cluster randomized controlled intervention follow-up study. In phase I of the study, the primary care health centers in the municipality of Bergen, Norway were randomly allocated to intervention or control groups. The intervention was carried out using the motivational interviewing technique and the common risk factor approach. The intervention group received guidance on diet/oral hygiene and the control group received standard care information. Parental knowledge and children at age 3 and 5 years old will be assessed in a prospective phase II follow-up study compared to native Norwegian controls. The primary outcome will be evaluation of change on parental oral health related knowledge and attitudes. The secondary outcome will assess the impact of the intervention on children’s caries -, body mass index- and oral health related quality of life.

**Discussion:**

Collaboration between dental public health and primary health care personnel on the common risk approach motivational intervention offers opportunities to address key dietary behaviors that may prevent obesity and dental caries. Providing sustainable preventive measures decreases the burden of diseases and consequently reduces health inequalities, particularly among at-risk children.

**Trial Registration:**

The study is registered as a clinical trial (ClinicalTrials.gov Identifier: NCT05758454: 7 March 2023). Ethical approval has already been granted by the Regional Ethical Committee (REK) (2015/ 27,639 /REK vest) and Sikt – Norwegian Agency for Shared Services in Education and Research (Reference number 778825).

**Supplementary Information:**

The online version contains supplementary material available at 10.1186/s12903-023-03329-9.

## Background

According to Statistics Norway [1], immigrants comprise nearly 20% of the population. They represent health-, societal- and economic assets, but also face challenges which the Norwegian society might help to address, facilitating their adaptation and integration to the new culture. A Norwegian of immigrant background is a person born in Norway, with one or two immigrant parents. Immigrants tend to find their changed living conditions disorienting, making it difficult for them to participate fully in the new society. For many reasons, a move to an unfamiliar environment may be difficult, not least due to lack of proficiency in the language of the new country. Several studies confirm that compared to the native population, the well-being of immigrants in Norway is poorer and they suffer more from lifestyle and diet-related disorders such as obesity [2–5].

Food security describes a situation in which there is physical, social, and economic access to safe, nutritious food, in quantities which meet the dietary needs and food preferences required to ensure an active and healthy life [6]. Food insecurity is reported to affect the well-being of children and adults of immigrant families in many Western countries, including the United States and United Kingdom [6, 7]. For most migrants, both the number of calories and the content of the diet change as a result of diverse factors, including language difficulties, lack of acculturation, shortages of previously used products, and culturally related factors associated with food. These factors underlying dietary changes may all contribute to different diseases, including a higher risk of obesity, type 2 diabetes, and cardiovascular disease as well as dental caries. Kumar and co-authors reported a high prevalence of obesity and overweight among immigrants from Pakistan and Turkey [5]. Studies primarily from the United States and United Kingdom have consistently shown that upon arrival, immigrants have lower BMI than their host-country-born counterparts. After longer residence, however, there is a change to higher BMI, overweight and obesity [8]. Compared with ethnic majority children in Denmark, Norway, and Sweden, non-western (NW) immigrant children show overall poorer health outcomes with respect to diabetes, obesity, oral health, mental health, and well-being [9]. A recent review of meta-analyses of data from studies conducted in developed and developing countries revealed that overweight and obese children had significantly more dental caries than children of normal weight [10]. In the sample population, both conditions were found to be associated with low levels of parental income and education [11]. Some immigrant groups who transition from a fiber-rich, whole grain diet to a high-fat diet with lower vegetable intake might be at increased risk of diabetes and cardiovascular disease [5]. A previous report has revealed that specific dietary habits (frequent intake of sugar and sweetened drinks) were considered to be common risk factors for both childhood obesity and caries [12].

In addition to dietary habits which constitute risk factors common to obesity and dental caries, other habits uniquely associated with heightened caries risk are common among immigrants. In some immigrant communities toothbrushing, oral hygiene routines and preventive dental care are less common and not regarded as important [13, 14]. Many families seek dental care only when a child is in pain or there is visible decay and preserving baby teeth may not be prioritized [15]. A Norwegian cohort study identified the following significant caries risk indicators among children at age 5 years: a mother consuming a diet very high in fat or sugar, a mother with low education and one or both parents of NW immigrant background [16].

Early childhood caries (ECC) is defined as any form of caries (cavitated or non-cavitated) occurring in the primary dentition of children aged 6 years or younger [17]. In children aged between 2.5 and 3.5 years, a combination of different factors constitutes predictors for developing caries, including the mother’s education, immigrant background, and consumption of candy and sugar-containing beverages [18].

In high-income countries there has been a marked decline in the incidence and prevalence of ECC over recent decades, reducing the psychosocial and economic burden of this disease for individuals and for society [19]. However, ECC remains an important health issue in particular population subgroups and in many countries, including Norway, immigrant children are found to be at high risk. Previous studies have revealed that children of NW immigrant backgrounds have higher caries experience than their native counterparts [17, 20–22]. This is attributed to lack of parental knowledge, poor communication due to language barriers and ethnic and cultural differences.

Children with ECC may experience pain when drinking hot or cold liquids, difficulty chewing and biting, poor appetite, weight loss, disturbed sleep, behavioral changes such as irritability and low self-esteem and a decline in academic achievement [23]. Furthermore, a recent study revealed that ECC has a negative effect on the quality of life (QoL) of preschool children and their families [22]. Further research is needed to determine whether and if so, to what extent, the quality of life of immigrant families living in Norway is affected by their children’s EEC.

Globally, obesity and dental caries constitute increasing public health problems. Both conditions are multifactorial, chronic, and highly prevalent, with significant and potentially lifelong impacts on health and quality of life [24]. The two conditions are thought to share common contributing factors, including biological, genetic, socioeconomic, cultural, environmental, dietary, and lifestyle factors [25]. The dietary factors include poor food choices, unhealthy dietary habits, frequent and high consumption of fermentable carbohydrates, consumption of sweetened foods/drinks, and high-calorie/cariogenic diets [26]. In a previous study, obesity, socio-economic factors, infrequent toothbrushing habits, and consumption of cariogenic beverages were identified as significant caries risk factors. It is of interest to note that a weak association was found between caries and extreme obesity (or morbid obesity) in children aged 4–17 years [20].

Though no evidence emerged of an association between obesity and caries among 6-year-old British children living in Plymouth, the deprivation highlights the vulnerability of children living in disadvantaged families to have both excess weight and more carious lesions [27].With caries and obesity acknowledged as the two most prevalent diseases of childhood, additional research should focus on the benefits and feasibility of widespread interdisciplinary medical-oral health collaboration in addressing and modifying common risk factors [28]. There are few culturally tailored health interventions. The fact that there are risk factors common to several systemic and oral health conditions, would support an approach in which oral health is integrated into primary health care services.

Reduction of free sugars in the diet can be part of a strategy to reduce risk factors common to both dental caries and childhood obesity. Thus, information about family standards, parental attitudes, and dietary habits of parents of NW immigrant backgrounds might provide a valuable tool for planning, implementation, and evaluation of culturally adapted health promotion programs. Intervention programs, focusing on dietary- and oral hygiene practices among NW immigrant families attending primary health care centers and based on a Common Risk Factor Approach (CRFA) and Motivational Interviewing (MI) might successfully reduce the incidence of caries and obesity. Such a program could be implemented within a community-based healthcare system throughout the country. Potentially, the trial results might be extrapolated to fill a knowledge gap about immigrant parents in Norway.

Several approaches for intervention have been applied to establish a model for child oral health promotion and to reduce inequalities in oral health. In a study carried out in Melbourne, families of Iraqi, Lebanese, or Pakistani backgrounds, with children aged 1–4 years, were invited to participate in an intervention project including community oral health education sessions and reorienting of dental health and family services. According to the authors, the findings from this study contribute to the limited evidence base in support of community interventions to improve child oral health in migrant communities [29]. In a recent study among Indian children aged 10–12 years, it was shown that an intervention in the form of oral health education (delivered through lectures and demonstrations by an undergraduate dental student) and topical antibacterial therapy (fluoride varnish and povidone-iodine) resulted in an improvement in caries and periodontal scores [30]. It is claimed that in indigenous communities in Alaska, community-centered multilevel interventions (educating families and communities and training mid-level dental care providers) will improve oral and systemic health and reduce pediatric tooth decay [31].

### Aims

The objectives of this project are:

1. To investigate the efficacy of a culturally adapted behavioral intervention on knowledge, attitudes, and behaviors related to a child’s risk for dental caries and obesity, with special reference to parents of NW immigrant background, with children aged 0–6 months.
2. To assess the effect of the behavioral intervention on children’s caries increment and family quality of life.
3. To generate research-based information about NW immigrants’ oral health issues, healthcare needs, and utilization of health services.
4. To evaluate possible differences and similarities in oral hygiene and feeding practices among parents of different cultural backgrounds (immigrants and native Norwegians).
5. To monitor parents’ attitudes to their children’s oral health in a follow-up study for children aged 3 and 5 years and to measure the children’s quality of life, body mass index (BMI) and caries status.

## Hypothesis

We hypothesize that compared to ethnic majority parents in Norway, immigrant parents have poor dietary knowledge, deleterious parental feeding habits and caring behaviors which might trigger caries and obesity among their newborn children. Small children of NW immigrant parents receiving common risk dietary interventions and guidance on hygiene/ feeding practices have better health parameters, including fewer carious lesions, standard BMI, and better quality of life than the control group.

## Methodology

### Trial Design

This is a cluster randomized controlled intervention follow-up study. By integrating primary health care and the public dental health service, the basis of the study is the application of an early-life intervention program among parents/children of immigrant backgrounds, using the MI technique and CRFA to prevent dental caries and obesity, thereby improving their general health, oral health, and quality of life. Primary care health centers in the municipality of Bergen, Norway, were randomly allocated to intervention or control groups (allocation ratio 1:1), to evaluate the efficacy of the culturally adapted dietary/hygiene advice. The intervention group received guidance on diet/hygiene and the control group received standard care, including regular health information at the primary care health centers. The study has been funded by the Norwegian Research Council for three years.

**Fig. 1:**
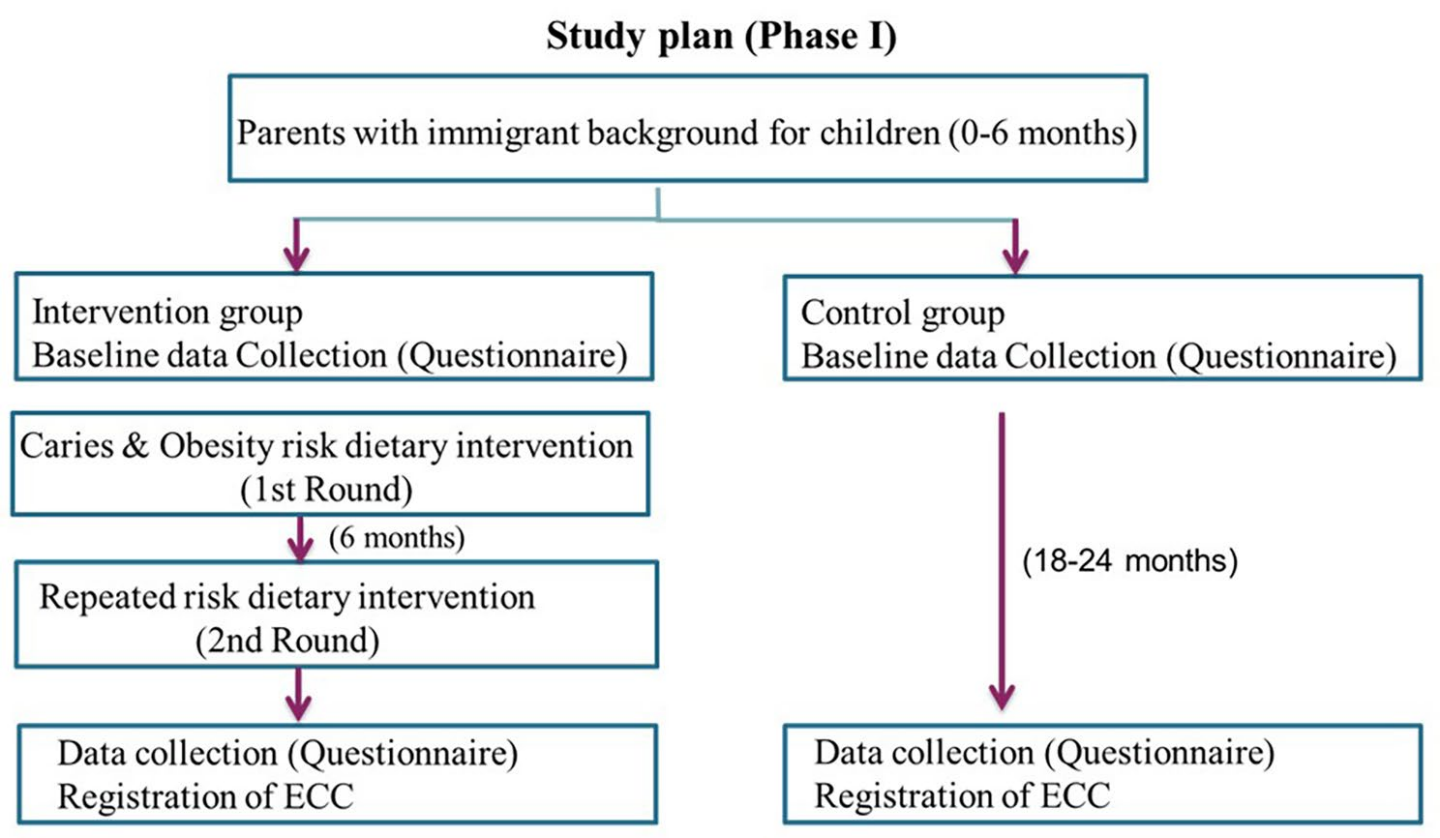
Study plan for phase I

The study has two phases:

**Phase I (The Intervention Study)** involves assessing oral health-related knowledge, attitudes, and behavior among immigrant parents of infants (0–6 months). It also involves investigating the potential of a culturally adapted oral health education intervention to prevent the development of ECC and to improve parental oral health-related knowledge and awareness (Fig. 1).

**Fig. 2:**
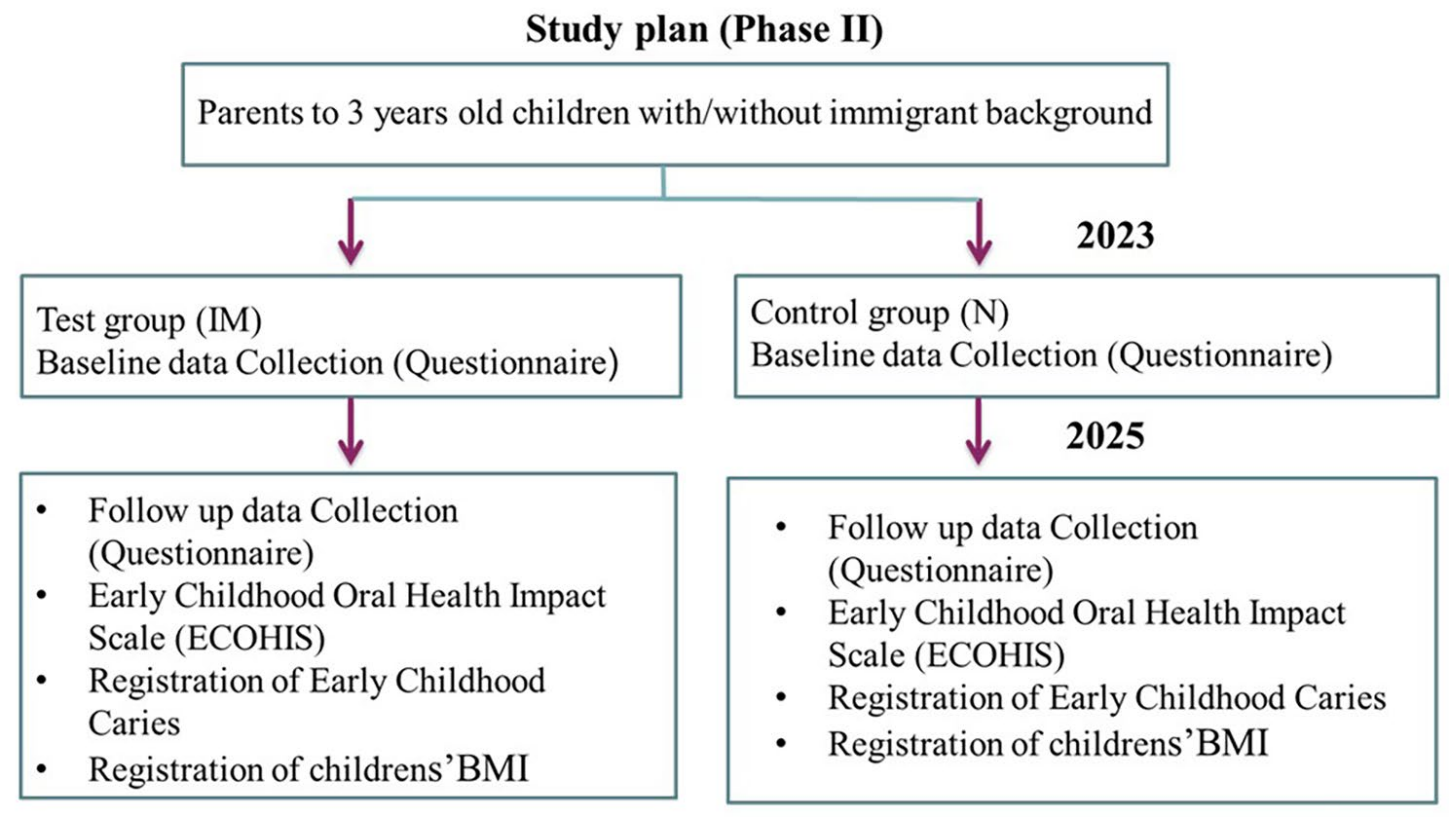
Study plan for phase II

**Phase II (follow-up Study)** involves following and comparing behavioral and attitudinal changes towards oral health and oral health-related quality of life among parental groups from different cultural backgrounds (immigrants, previously involved in phase I and native Norwegians), It also includes BMI measurements of children, and caries status in both the test group (immigrant, IM) and the control group (Norwegian, N) over a two-year period. (Fig. 2).

### Study population

Parents were recruited from the municipality of Bergen, Norway.

### Inclusion criteria

#### Phase I

Immigrant parents (IM) of infants (0–6 months old) with NW background (Eastern Europe, Asia, Africa, Turkey, South and Central America) were recruited from different primary health care centers in the municipality of Bergen, Norway. Before the recruitment phase, the health centers were allocated to either intervention or control groups.

#### Phase II

The immigrant parents involved in the intervention study (IM) and a new group of parents of Norwegian background (N) will be recruited from similar public dental health clinics when they visit these centers for their children’s routine oral health examinations at the ages of 3 and 5 years.

### Exclusion criteria

Parents who were unwilling to participate or whose babies had serious congenital defects, requiring frequent oral health checks, were excluded.

### Screening and recruitment process

**Fig. 3:**
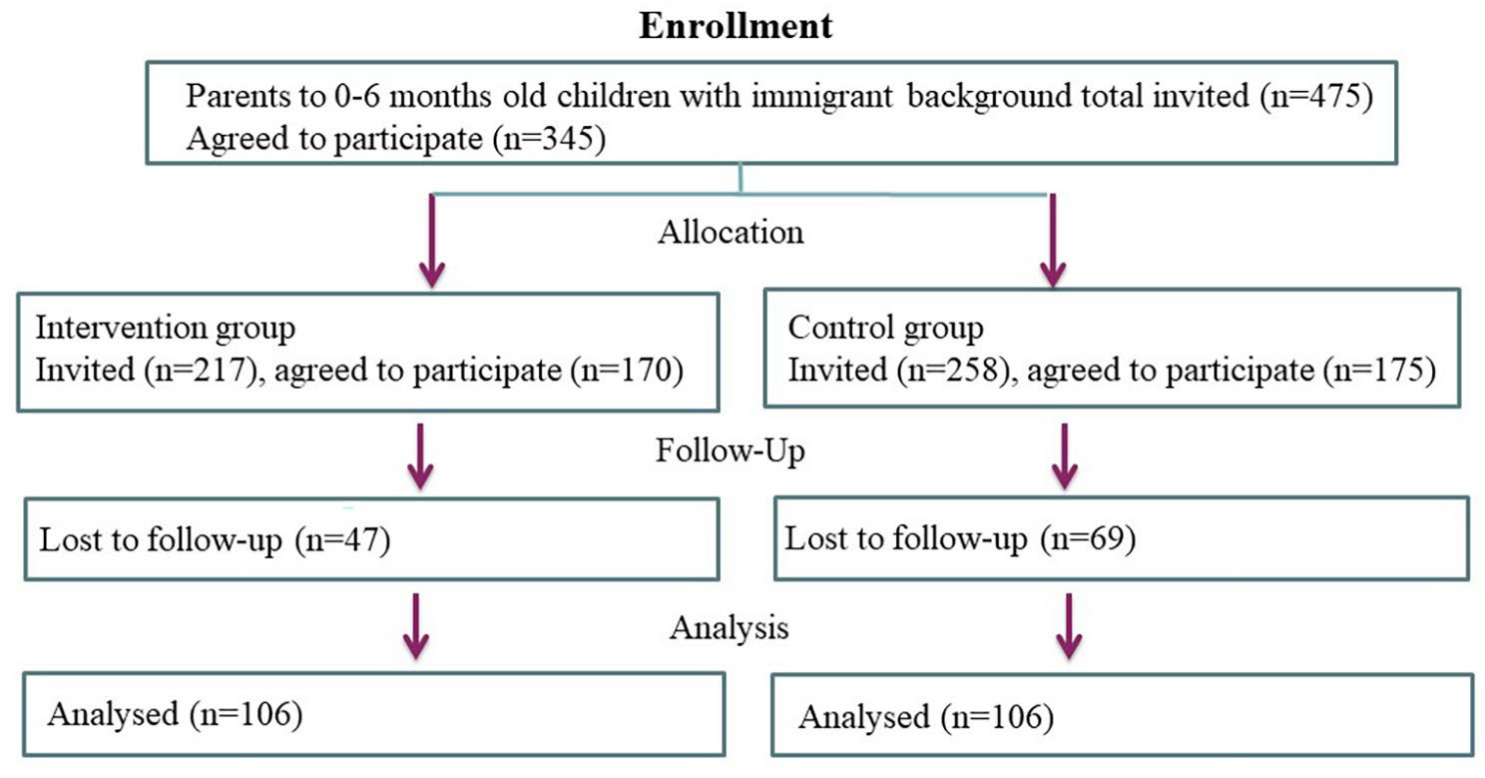
Consort diagram. This figure illustrates the study design (RCT). A total of 7 health centers having 475 participants with children 0-6 months were randomized to an intervention arm and a control arm. RCT, randomized controlled trial

The health centers selected for participation have different uptake areas within the municipality of Bergen and have high numbers of parents of immigrant background enrolled. Before vaccination, the medical staff of the primary health care centers briefed the parents about the project and those who were interested in learning more about the study were directed to a room where they received further details from the researchers involved in the project. The researchers were responsible for handling requests to participate in the study, information about the study and handing out the consent form. During waiting time and after the child’s vaccination, personal interviews were conducted with those parents who were willing to participate/sign the consent form (Fig. 3).

While parent and child waited for the recommended time before discharge after vaccination, an experienced dental professional researcher explained the purpose of the study and provided participating immigrant parents (IM) with the interview schedule. The intervention group received additional information on oral hygiene, modifying dietary risk behaviors, and dietary guidance, while the control group received the regular health information provided at the primary care centers.

Strategies to promote retention include provision of health information. The family was issued with a toothbrush for the child and a plastic mouth mirror for the parent to check the oral cavity.

The child included in the study was referred for dental treatment if needed (the child’s first routine visit to the dentist is at 3 years of age). Reminders about upcoming interviews were sent to the participants and confirmed by telephone.

### Sample size

**Fig. 4:**
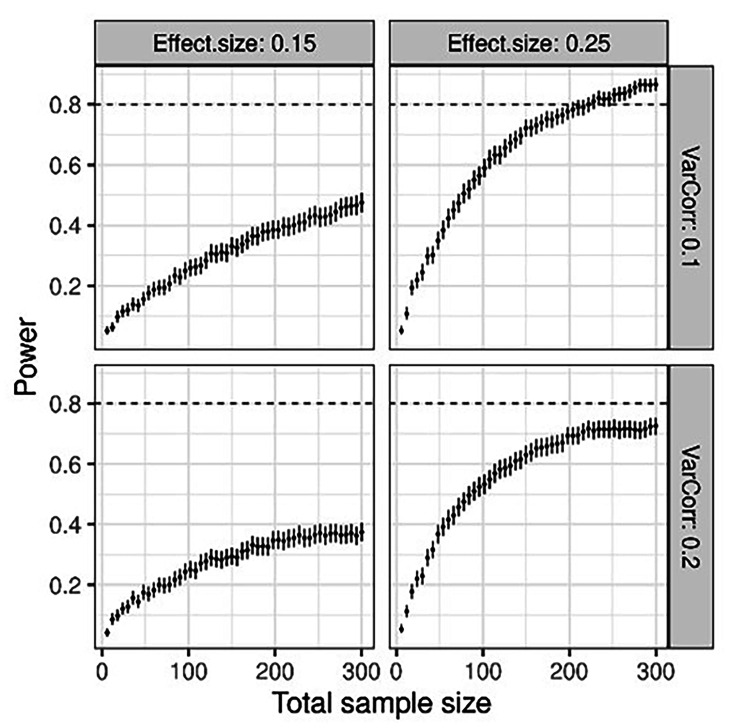
Sample size

Statistical power (+/- 95% CI) to detect a fixed effect the size of 0.15 or 0.25 (left and right panels, respectively) and with a random effect of clusters (health stations), measured in standard deviations, was set to 0.1 or 0.25 (top and bottom panels, respectively). Power was calculated over a range of sample sizes using the power Curve function from the *simr* R library. The number of clusters (health stations) is six, i.e., three per treatment group. Each data point and associated confidence interval are estimated from 1000 Monte Carlo simulations. The results indicate that a total sample size of at least 300 participants (approximately 50 parents/child pairs from each center) is needed to ensure adequate power, given that the effect size is no smaller than 0.25 (Fig. 4).

### Randomization

Health centers in the municipality of Bergen (i.e., health centers agreeing to participate in the study) were randomly allocated to intervention or control groups by researchers involved in the study. Instead of randomization of individuals within health centers, cluster randomization of the primary sampling unit (health centers) was deemed necessary to avoid contamination of information from those in the intervention group to those in the control group while sharing the waiting room of the same health centers.

### Data collection and assessment

#### Data collection instrument

Data will be collected in both phase I and phase II, using a questionnaire intended to elicit parental information about caries and obesity risk behaviors as well as behavioral determinants that will be targeted during the intervention.

The ***first*** part of the interview schedule will assess demographics and socioeconomic status. Demographic data include age, gender, and indicators of socioeconomic and migration status: father’s-mother’s education, occupation, and years of residence in Norway. The ***second*** part of the interview schedule will include parental *knowledge related to* breastfeeding, healthy feeding practices, etiology and prevention of obesity, and dental caries, *attitudes* (food habits, tooth cleaning, regular dental visits), eating habits (sugary food/drinks intake, frequency of consumption of sweet beverages) and different oral hygiene practices. The theory of planned behavior (TPB) will be operationalized according to the practices of the immigrant parents. The ***third*** part of the interview schedule will include information about the parents’ own oral hygiene and dietary habits, cultural traditions, perceived oral health and the utilization of general/dental health services. Finally, the Early Childhood Oral Health Impact Scale (ECOHIS) will be used to measure the quality of life of the children and their families. The ECOHIS is a tool used to analyze the impact of oral health issues on the quality of life of families with children aged between 3 and 5 years [32].

### Outcomes

#### Primary outcome measures

**Change in poor parental knowledge of children’s oral health**

The questionnaires completed by IM before and after a culturally adapted informational intervention will be used to measure the change in parental knowledge. Responses of parents to the first questionnaire, when the child is 0–6 months, and then when the child is 18–24 months were collected. The changes between and within the control and intervention groups will be evaluated.

**Change in poor parental behavior regarding children’s oral health**

The questionnaires completed by IM before and after culturally adapted informational intervention were the basis for measuring the change in parental behavior. Similarly for this outcome, the changes between and within the control and intervention groups will be assessed.

#### Secondary outcome measures caries increment

(a) Clinical examination of caries lesions in the maxillary anterior teeth when the child is 24 months of age. Examination at 6 months is not possible because at this age no teeth have yet erupted. The comparison will be carried out between and within the control and intervention groups. Therefore, examination (duration ~ 15 min) was carried out when the child was 24 months. (b) Caries increments between the ages of 3- and 5-years will be registered from public dental health clinic records. The outcomes for control (N) and the test (IM) groups will be compared.

**Body Mass Index (BMI)**

The BMI of the child will be determined by measuring the height (meter) and weight (Kg) when the child is 5 years old. The control (N) and the test (IM) groups will be compared.

**Quality of life of children /families**

The quality of life of children/families will be measured using a validated instrument (ECOHIS), including a child and family component. The responses of parents to the first questionnaire when the child is 3 years, and when the child is 5 years of age will be collected. The control (N) and the test (IM) groups will be compared.

### Operational framework

#### The intervention

The intervention (oral health education) was provided in the form of individual presentations of about 30 min. Thereafter, a practical demonstration of tooth brushing was presented. Each participant received a carry-home message in the form of a pamphlet containing similar information to the presentation, in the participants’ mother tongue. Apart from brushing, there is information about the primary dentition, dietary habits and their effect on caries and obesity. Bottle feeding and its effect on ECC was emphasized. Moreover, the importance of regular dental visits was highlighted. A pilot study of 20 immigrant parents had been conducted previously, to assess the questionnaire to be used and the intervention to be applied. The control group will be provided with the intervention material after the end of the study.

#### Theoretical framework

Different theoretical approaches are used in this project, including:

MI: defined as “a person-centered counseling style for addressing the common problem of uncertainty about change”. A recent systematic review identified MI as being the most effective approach for altering health behavior in an individual oral health promotion setting [33]. Therefore, it is strategic to use culturally adapted MI to convey actionable information and motivate health-related behavioral change in families with newborn children.

CRFA: This approach addresses risk factors common to many chronic conditions within the context of the broader socio-environmental setting [26]. The main implication of CRFA for oral health policies is collaboration with other sectors and disciplines. Oral health issues need to be integrated into primary health care, with recommendations which promote general health. As some chronic diseases share common causes, adopting a collaborative CRFA is more rational than focusing on one disease.

TPB: is a commonly utilized social cognitive theory, predicting behavioral intention and behavior. TPB incorporates three main constructs that influence behavioral intention, including attitude, subjective norms, and perceived behavioral control. The TPB has successfully accounted for a high proportion of variance in several health-related behaviors including those related to oral health [34]. In this project, we will use the TPB to explain dietary behavior intentions of parents with NW immigrant background and identify the most important causal factors. Dietary intention is predicted by attitudes, subjective norms, and perceived behavioral control. According to the TPB, the effectiveness of educational strategies depends on successfully changing the most important attitudinal components of TPB. The relative weight of the predictors of behavioral intention determines whether the main focus should be on attitudes, subjective norms, or perceived behavioral control.

### Statistical analysis

The data will be analyzed using STATA and SPSS version 24 (SPSS Inc., Chicago, II, USA). Descriptive statistics such as mean and standard deviation for continuous variables and frequency and percentage for categorical variables will be presented. Univariable analysis will be based on cross tabulation and Chi Square statistics. Independent sample t test will be used to assess how sum scores of knowledge, attitudes and the parent’s own oral hygiene and dietary habits varied according to the parents’ age, educational level, and duration of residence in Norway. Differences between intervention and control groups will be analyzed using T-tests and associations will be quantified using eta coefficients. A two-sided significance level of 5% will be applied in all analyses.

Primary analysis will follow an “Intention-to-treat” approach. Comparison between the intervention and control groups at baseline and follow-up to calculate intragroup changes of outcome variables will be performed using generalized linear model (GLM) repeated measures. In the secondary analysis, ‘per-protocol’ approach will be used, where the participants would be excluded if they (1) did not participate in the intervention program, (2) did not complete the questionnaires, (3) were not eligible according to the inclusion and exclusion criteria.

## Discussion

Parents of immigrant background are not able to derive much benefit from the health information currently provided at healthcare centers. This is attributed to low attendance rates, poor skills in the host language and illiteracy in terms of beliefs and behaviors which differ from those of the host population [35]. A previous Norwegian study suggests that immigrant groups in Western societies require different information packages, modified strategies, and encouragement to exercise discipline with reference to factors which have deleterious effects on general and oral health [36]. Thus, this work indicated that NW immigrant families with small children might be in great need of an early, culturally adapted intervention in the form of healthy dietary advice/guidance and early oral health care. Currently there is scarce evidence to support the efficacy of culturally tailored health interventions (interventions which take culture-specific factors into account).

### Need for intervention

Why use MI and CRFA in the present innovative intervention?

To reduce common dietary-related behavior, which contributes to the risk of obesity and dental caries in children, parents need to be educated about the sugar content of processed foods and beverages as well as the negative impact on their children’s health of frequent daily sugar consumption. Moreover, health professionals must engage in identifying children who are at risk for obesity and dental caries and provide education, screening, and referral to reduce common risk behaviors [37]. Furthermore, by engaging primary healthcare professionals, this project might contribute to solutions for different challenges, including communication when encountering parents of immigrant background seeking health and welfare services. By creating a collaborative model between primary care and dental health services, the project prioritizes services which are sustainable and cost-effective for society, resulting in user satisfaction.

This outcome has been documented in previous reports. In an Australian study, families of Iraqi, Lebanese, or Pakistani background were offered community oral health education sessions and reorienting of dental health and family services, with promising results [25]. Ninty three percent of caregivers of children aged 6–36 months reported positive behavioral changes after an intervention to improve feeding practices. The authors of this study stated that partnerships between dental and nutrition professionals offer opportunities to address key dietary behaviors that may prevent obesity and improve oral health, particularly among children at risk [11]. While intervention studies focusing on diet-related diseases in young children have reported various outcomes, MI has proved to be the most effective [38].

The main strength of this study is that it is a randomized trial, making it possible to evaluate the effectiveness of an intervention program to prevent caries and obesity. The use of cluster randomization allows the identification of the intervention effects when randomization at individual level is not appropriate. Cluster randomization may help to prevent contamination caused by sharing of information between intervention and control participants. Using a culturally adapted health intervention program, focusing on dietary- and oral hygiene practices among NW immigrant families attending primary health care centers might positively impact parental attitudes towards the oral health of their children. In this study, the collaboration between dental public health and primary health care personnel using the CRFA and MI is an innovation which offers opportunities to address key dietary behaviors that may improve general and oral health, particularly among at-risk children.

A possible limitation of this study is that data will be collected by means of a self-reported questionnaire, in which participants might be inclined to give more socially acceptable answers. The diverse backgrounds and different periods of residence in Norway might influence comparisons between groups as well as the acculturation effects. Finally, the two sessions for data collection limit the potential to draw causal inferences about the influence of parental characteristics on their hygiene/feeding practice knowledge and attitudes.

### Dissemination, sharing and utilization

We intend to disseminate the findings to the professionals most closely involved (e.g., dentists, primary care health personnel, etc.) and to extend this information to other communities in order to increase general awareness of the health care needs of immigrants and to foster interdisciplinary collaboration between oral and primary health care authorities. To improve health awareness among immigrants, educational seminars targeting newly arrived immigrants will be arranged at local health service centers, including public primary health care centers, dental public health service centers and health information days arranged by dental students. In this context the informative intervention materials produced for the present study will be of help to the primary health care medical staff to improve communication challenges with NW immigrant parents.

### Trial Status

Recruitment started in 2019,was continuing when the manuscript was submitted and is estimated to finish in 2025.

### Electronic supplementary material

Below is the link to the electronic supplementary material.

Supplementary Material 1. Additional file 1. SPIRIT Checklist

Supplementary Material 2. Additional file 2. SPIRIT Figure

Supplementary Material 3. Additional file 3. Items of the questionnaire

## Data Availability

The study data used will be available from the corresponding author (MM) upon request.

## Declarations

### Confidentiality

Identification numbers without names will be used to guarantee the participant’s confidentiality during data analysis. In addition, participant files will be stored in a secure location. Data protection clearance will be sought from the Norwegian Data Inspectorate.

### Data monitoring

Data collection, management, control, and analysis will be undertaken independently by MM.

### Harms

The project includes a questionnaire for the parents and caries/BMI’s registration of young children, so the disadvantages are small, as described under the individual project participants. There is therefore no physical, psychological, social and/or practical risk/damage/discomfort/burden now or in the future for the participants, society and/or the environment.

### Auditing

The data entered will be managed by the study authors. Data will be reviewed periodically. Any discrepancies will be examined, fixed, and recorded.

## List of abbreviations

BMI: Body mass index
CI: Confidence Intervals
CRFA: Common Risk Factor Approach
ECC: Early Childhood Caries
ECOHIS: Early Childhood Oral Health Impact Scale
IM: Immigrant
MI: Motivational Interviewing
N: Native Norwegian
NW: Non-western
QoL: Quality of Life
TPB: Theory of Planned Behavior
