# Correction: Prevention of caries and obesity in children with immigrant background in Norway- a study protocol for a cluster randomized controlled trial

**DOI:** 10.1186/s12903-023-03616-5

**Published:** 2023-11-27

**Authors:** Mariam Reda, Abhijit Sen, Anne-Kristine N. Åstrøm, Manal Mustafa

**Affiliations:** 1 Oral Health Centre of Expertise in Western Norway, Bergen, Norway; 2 Department of Clinical Dentistry, Faculty of Medicine, University of Bergen, Bergen, Norway; 3 Center for Oral Health Services and Research (TkMidt), Trondheim, Norway; 4 Department of Public Health and Nursing, Faculty of Medicine, and Health Sciences, Norwegian University of Science and Technology, Trondheim, Norway

**Correction: BMC Oral Health 23:620 (2023)**

**https://doi.org/10.1186/s12903-023-03329-9**

In this article [1], co-author Anne-Kristine N. Åstrøm is omitted in the source file received for production and overlooked in subsequent stage as well.

Anne-Kristine N. Åstrøm is the third author in the author group, and affiliated with Department of Clinical Dentistry, Faculty of Medicine, University of Bergen, Bergen, Norway.
