# Supplementary material for: Prevention of caries and obesity in children with immigrant background in Norway- a study protocol for a cluster randomized controlled trial
Source: BMC Oral Health. 2023 Sep 1;23:620. doi: 10.1186/s12903-023-03329-9 (PMC10474737; doi:10.1186/s12903-023-03329-9)
Supplement: Supplementary file 2 — Supplementary Material 2. Additional file 2. SPIRIT Figure [file 12903_2023_3329_MOESM2_ESM.docx]

Figure. SPIRIT Schedule of enrolment (Phase I), interventions, and assessments

|  | **STUDY PERIOD** | | | |
| --- | --- | --- | --- | --- |
|  | **Enrolment** | **Allocation** | **Post-allocation** | |
| **TIMEPOINT**** | ***-T_1_*** | **T*_0_*** | ***T_1_*** | ***T_2_*** |
| **ENROLMENT:** | X |  |  |  |
| **Eligibility screen** | X |  |  |  |
| **Informed consent** | X |  |  |  |
| **Randomization by independent researcher** | X |  |  |  |
| **Allocation (after baseline assessments)** |  | X |  |  |
| ***Intervention:*** |  |  |  |  |
| **ASSESSMENTS:** |  |  |  |  |
| ***[Demographic and background variables]*** | X | X |  |  |
| ***[Primary outcome variables: Parental oral health related knowledge and attitudes*** |  | X | X | X |
| ***[Secondary outcomes:***  ***Childhood Caries]*** |  | X | X | X |
| ***[Process evaluation]*** |  |  |  | X |
