# Supplementary material for: Prevention of caries and obesity in children with immigrant background in Norway- a study protocol for a cluster randomized controlled trial
Source: BMC Oral Health. 2023 Sep 1;23:620. doi: 10.1186/s12903-023-03329-9 (PMC10474737; doi:10.1186/s12903-023-03329-9)
Supplement: Supplementary file 3 — Supplementary Material 3. Additional file 3. Items of the questionnaire [file 12903_2023_3329_MOESM3_ESM.pdf]

Interview guide

Structured interview schedules will be used by trained interviewers to assess parents' oral health related knowledge-, attitude and behavior. Standardized questionnaires translated to Norwegian will be used by the interviewers at baseline 0-6 months after birth and at 18-24 months follow up in the first intervention part of this project. This questionnaire will be modified and implemented further among immigrant parents/children and Norwegian born parents/children in a (control group) in a prospective follow up at children aged 3 (2023) and 5 (2025). In the prospective cohort study of immigrants and Norwegian controls, the questionnaire will be extended with questions regarding children's own eating behavior and other oral health behaviors relevant for oral health. At age 5 (2025), oral health related quality of life will be assessed using the Early Childhood Oral health Impact Scale (ECOHIS) inventory, validated in various cultural contexts (ref). Face to-face questionnaire-based interviews will be administered at each time point of data collection. The interviewers should be bilingual (Norwegian/English). Interpreter will be used if parents are willing to participate but they cannot communicate in Norwegian or English. (See attached the extended questionnaire schedule used in the prospective cohort study among immigrants and controls).

Questionnaire content for immigrant parents and Norwegian control parents

"Attitude to Hygiene"

As a family we intend brushing our child's teeth for him/her

We intend brushing our child's teeth for him/her twice a day

The people in my family would feel it was important to help brush child's teeth twice a day

The people we know well would feel it was important to brush our child's teeth twice a day

We feel able to brush our child's teeth for him/her

I don't know how to brush my child's teeth properly

If we brush our child's teeth twice a day, we can prevent our child getting tooth decay

If our child uses a fluoride toothpaste, it will prevent tooth decay

We don't have time to help brush our child's teeth twice a day

We cannot make our child brush his/her teeth twice a day

"Attitude to Diet"

As parents it is easy for us to control children' sugar consumption

As parents it is difficult for us to control children' sugar consumption

As a family, we intend controlling how often our child has sugary snacks between meals

The people in my family would feel it was important to control how often our child has sugary foods and drinks between meals

“Parental Indulgence”

It is worthwhile to give our child sweets/biscuits to behave well

In our family, it would be unfair not to give sweets to our child every day

It is often too stressful to say “no” to my child when he/she wants sweet

If our child does not want to brush his/her teeth daily, we don’t feel we should make them

It is not worth it to battle with our child to brush his/her teeth twice a day

oral health related behavior

Parents

How often do you usually brush your own teeth?

How often do you usually use toothpicks / dental floss?

How often do you usually use fluoride rinse or other mouthwash?

How often do you usually eat sweets / snacks?

How often do you usually drink sweet drinks (Tea/ coffee with sugar / cola / soda / juice)?

How often have you been to the dentist in the last 5 years?

How do you assess your own oral health?

children

How often your child usually brushes his/her teeth?

Does your child brush with fluoride toothpaste?

How often does your child usually eat sweets / snacks?

Does your child eat sugary snacks between meals?

Children’s oral health related quality of life (ECOHIS)

Child impact- Has child:

Ever had toothache

Ever had swollen/bleeding gums

Ever cried because of pain in mouth

Ever failed to sleep because of pain in mouth

Ever refused to eat because of pain in mouth

Ever refused to play

Family impact

How often have you or another family member because of problems with child’s mouth and teeth?

Taken time off work

Been upset

Felt guilty

Had financial difficulties
